# Ethics, Integrity, and Retributions of Digital Detection Surveillance Systems for Infectious Diseases: Systematic Literature Review

**DOI:** 10.2196/32328

**Published:** 2021-10-20

**Authors:** Ivy Y Zhao, Ye Xuan Ma, Man Wai Cecilia Yu, Jia Liu, Wei Nan Dong, Qin Pang, Xiao Qin Lu, Alex Molassiotis, Eleanor Holroyd, Chi Wai William Wong

**Affiliations:** 1 WHO Collaborating Centre for Community Health Services School of Nursing The Hong Kong Polytechnic University Hong Kong SAR China; 2 Department of Family Medicine and Primary Care Li Ka Shing Faculty of Medicine The University of Hong Kong Hong Kong SAR China; 3 Shenzhen Institute of Advanced Technology Chinese Academy of Sciences Shenzhen China; 4 Department of Information Technology University of Hong Kong-Shenzhen Hospital Shenzhen China; 5 School of General Practice and Continuing Education Capital Medical University Beijing China; 6 School of Clinical Sciences Auckland University of Technology Auckland New Zealand; 7 Department of Family Medicine and Primary Care University of Hong Kong-Shenzhen Hospital Shenzhen China

**Keywords:** artificial intelligence, electronic medical records, ethics, infectious diseases, machine learning

## Abstract

**Background:**

The COVID-19 pandemic has increased the importance of the deployment of digital detection surveillance systems to support early warning and monitoring of infectious diseases. These opportunities create a “double-edge sword,” as the ethical governance of such approaches often lags behind technological achievements.

**Objective:**

The aim was to investigate ethical issues identified from utilizing artificial intelligence–augmented surveillance or early warning systems to monitor and detect common or novel infectious disease outbreaks.

**Methods:**

In a number of databases, we searched relevant articles that addressed ethical issues of using artificial intelligence, digital surveillance systems, early warning systems, and/or big data analytics technology for detecting, monitoring, or tracing infectious diseases according to PRISMA (Preferred Reporting Items for Systematic Reviews and Meta-Analyses) guidelines, and further identified and analyzed them with a theoretical framework.

**Results:**

This systematic review identified 29 articles presented in 6 major themes clustered under individual, organizational, and societal levels, including awareness of implementing digital surveillance, digital integrity, trust, privacy and confidentiality, civil rights, and governance. While these measures were understandable during a pandemic, the public had concerns about receiving inadequate information; unclear governance frameworks; and lack of privacy protection, data integrity, and autonomy when utilizing infectious disease digital surveillance. The barriers to engagement could widen existing health care disparities or digital divides by underrepresenting vulnerable and at-risk populations, and patients’ highly sensitive data, such as their movements and contacts, could be exposed to outside sources, impinging significantly upon basic human and civil rights.

**Conclusions:**

Our findings inform ethical considerations for service delivery models for medical practitioners and policymakers involved in the use of digital surveillance for infectious disease spread, and provide a basis for a global governance structure.

**Trial Registration:**

PROSPERO CRD42021259180; https://www.crd.york.ac.uk/prospero/display_record.php?RecordID=259180

## Introduction

In the wake of the global COVID-19 outbreak, there is growing pressure to improve our existing practice in the prevention and ongoing monitoring of emerging infectious diseases and the adoption of targeted interventions for emerging infectious diseases. Current infectious disease surveillance systems in most countries are remarkably similar. Once a case is clinically suspected and confirmed, there are multiple levels of reporting [1]. Then, the accumulated information from local institutions is aggregated, processed, and defined at the population level before actions are subsequently disseminated through the system from a “top-down” approach. The existing process carries an inevitable time lag that can result in both reduced effectiveness for responsive public health interventions [2] and opportunities for doctors and patients to negotiate reporting, which can have catastrophic results, as had been observed in the early COVID-19 outbreak in China [3] and the Ebola outbreak in West Africa [4]. The need for timely data collection or sharing, processing, decision making, and reporting in infectious disease surveillance has been identified as one of the main drivers for introducing artificial intelligence (AI) technology.

With the establishment of electronic health records (EHRs), big data have been acquired, making it possible to build data-intensive infectious disease surveillance or early warning systems, pre-empt emergency response, and strengthen infection prevention and control. Machine learning (ML) technologies, a multiplying form of AI, has shown considerable potential in tracing the source and detecting potential outbreaks or novel infectious diseases using patients’ EHRs. By utilizing real-time digital data analysis, a fully automated system could be built to transmit, through extraction, structured data and doctors’ medical records in text, while new technologies, such as named entity recognition, would allow extraction of patient-related features from the unstructured text into predefined categories to support future infectious disease monitoring and surveillance [5]. Examples of enhanced timeliness resulting from this approach in COVID-19 case tracing have been reported in China and several other Asian counties [3]. When AI is being employed for infectious disease control (eg, using mobile phone apps to trace COVID-19 cases), potentially infected patients, their close contacts, and, at times, larger communities can be tracked, tested, and, if necessary, quarantined to prevent further outbreaks.

Nonetheless, the advantages of big data and ML in infectious disease control need to be weighed against the considerable ethical and legal concerns pertaining to the protections and privacy of individuals and the public in respect to access, use, and sharing of large data sets of patients’ medical records. These kinds of AI interventions raise complex contemporary ethical questions regarding potential misuse of personal information and informed consent that have the potential to infringe on one’s human and civil rights. Furthermore, there is the heightened risk of patients’ personal information being leaked to social media when they have previously been assured of confidentiality and privacy [6]. Reidentification of named patients is a major concern when databases are hacked [7], and data custodians may sell data for financial gain to pharmaceutical, insurance, or software companies [8]. Furthermore, the routine use of big data analytics (BDA) or the ethics and widespread moral implications of ML continue to be vigorously debated around the accuracy of reporting [9,10], and the consequences of inaccuracies in the reporting of outbreaks are gaining considerable attention [11]. These developments, often in rapid response situations, have sparked issues between optimizing population health outcomes informed by epidemiology and public health, and societal or individual ethical rights and protections that inform human rights and freedom of choice.

Notwithstanding these debates, BDA is critical for managing communicable disease spread or outbreaks in today’s digital world where consistency in the application of regulations or rules of privacy, confidentiality, transparency, data handling, and security safeguards for ML transmission required to protect individuals’ rights is limited [12]. This study set out to examine the ethical issues of using AI and identify an intersection or balance between the protection of individuals’ human rights and patients’ autonomy, and common good for population-based public health outcomes specific to infectious disease control and prevention with the aim of investigating ethical issues identified from utilizing AI-augmented surveillance or early warning systems to monitor and detect common or novel infectious disease outbreaks.

## Methods

### Search Strategy and Selection Criteria

We searched broad search engines with no time restriction. CINAHL, PubMed, Science Direct, MEDLINE, Google Scholar, and Scopus, as well as legal and sociological databases were searched from database inception to December 8, 2020. Additionally, relevant articles from the documents’ bibliography and from other articles that cited the documents were retrieved. The following search terms and combination of terms were utilized: ethic* OR “data security” OR “data privacy” OR sensitiv* OR confidential* OR anonym* OR “personally identifiable information” OR privacy or “human right*” AND “electronic health records” OR ehr OR “Clinical decision support system” OR cdss OR “Artificial Intelligence” OR ai OR “augmented surveillance” OR surveillance OR “contact tracing” OR “Machine Learning” OR ml OR “deep learning” AND “Infectious Disease” OR “Communicable Diseases.” Prior to the searches, the authors reviewed all search terms. In order to identify all existing literature for this review, we considered all peer-reviewed empirical research articles, review reports, and grey reports. To enhance the rigor of the review, our approach followed the PRISMA (Preferred Reporting Items for Systematic Reviews and Meta-Analyses) guidelines (Multimedia Appendix 1) [13].

### Inclusion and Exclusion Criteria

There were no language restrictions placed on the literature search. To be included for further review, the collected articles must have addressed the ethical issues of using AI, digital surveillance systems, early warning systems, and/or BDA technology for detecting, monitoring, or tracing specifically infectious diseases.

### Literature Selection

**Figure 1:**
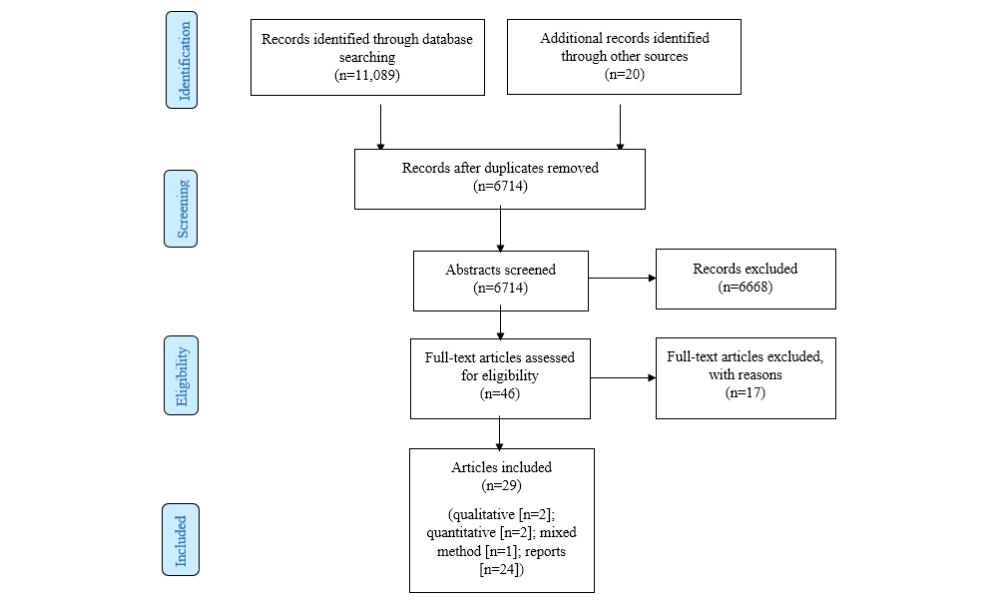
PRISMA (Preferred Reporting Items for Systematic Reviews and Meta-Analyses) flowchart of the literature review.

We selected literature for inclusion in 2 stages to ensure rigor. In the first stage, 2 authors (IYZ and YXM) independently screened the titles and abstracts of all citations for potentially relevant articles. In the second stage, the same 2 authors independently examined the full texts of these papers against prespecified inclusion criteria. Any discrepancies were resolved with input from a third author (EH). Of the 6714 titles and abstracts reviewed, we excluded 6668 articles that did not meet our eligibility criteria, resulting in 46 articles for full-text review. Additionally, 17 articles were excluded because they (1) were not related to digital surveillance systems, (2) did not focus on infectious diseases, or (3) lacked focus on ethical concerns. In all, 29 papers satisfied our eligibility criteria and were included by consensus agreement (Figure 1).

### Data Extraction, Quality Assessment, and Data Analysis

All authors read and reviewed the 29 articles in order to summarize the approaches, methodologies, samples, and findings. Two authors (IYZ and YXM) undertook literature quality assessment, extracted data from the literature, included them into a spreadsheet, and analyzed the data independently. Discrepancies were resolved, and data were confirmed in several rounds of discussions with other team members. We evaluated study quality and methodological rigor for 5 empirical studies by using the modified mixed-methods appraisal tool (MMAT) [14], which was not applicable for the remaining discussion papers. Lower quality scores did not result in exclusion of any articles. However, the findings of articles with lower quality scores were given less weight during data analysis.

We structured our identified ethical issues using a theoretical framework developed by Asadi et al [15], in which key concepts of prior BDA had been identified, defined, and examined using stakeholder theory and discourse ethics [16]. Key themes from our review were discussed and summarized, and the gaps in the literature and methodologies were identified.

## Results

### Overview and Participant Demographics

Among the 29 included articles, 2 qualitative studies [17,18], 2 quantitative studies [19,20], 1 mixed methods study [21], and 24 discussion papers were identified (Table 1). All of the selected articles were published between the years 2015 and 2021. The 5 empirical studies [17-21] adopted a combination of focus groups, a Delphi approach, a database digital ethnography, and surveys as research methods, and they were all identified as high quality. Together they included 7331 participants. Of these 5 studies, 1 was conducted in South Korea [21] and 4 in Australia [17,19-21]. Four studies [17-19,21] reported gender ratios and 3 [17,19,21] demonstrated age ranges. Research participants were the general public; policymakers; and experts in infectious diseases, epidemiology, food safety, health informatics systems, and health and technology law.

**Table 1:** Summary of ethical issues identified from utilizing digital surveillance systems for infectious diseases.

Article type, and authors and year			Country		Journal		1. Sampling frame 2. Sample size 3. Age 4. Gender ratio (F:M) 5. Sampling method		Study aim		Context of studies		Methodology and methods		Key and relevant findings
**Journal articles (n=5)**															
	Degeling et al (2020) [17]	Australia		BMC Medical Ethics		1. People who had previously volunteered to take part in research and topic-blinded social media advertising on Facebook 2. n=50 3. 18-34 y (n=15); 35-54 y (n=22); and >55 y (n=11) 4. 27:21 5. Random sampling		Examine the public acceptability and ethical concerns of community juries on integration of big data analytics (BDA) into communicable disease control		BDA was perceived as intrusive and a threat to privacy.		Qualitative; deliberative group sessions; Delphi study approach		Almost all jurors supported data linkage for public health research and suggested deidentification practices. Three juries raised several conditions related to system oversight and security being met. One concern was about loss of privacy and mistrust in governments to run secure and effective systems.	
	Kim et al (2021) [18]	Korea		Social Science & Medicine		1. Comments from January to May 2020 made by Korean mothers on 15 internet groups called “mom cafes” 2. n=3729 3. N/A^a^ 4. Female 5. Purposive sampling		Examine how Korean mothers understand morality in the context of COVID-19 contact-tracing surveillance		Korean mothers uploaded COVID-19 patient information on the boards of online groups for discussion.		Qualitative; database digital ethnography; reflexive thematic analysis		Nonmaleficence is the core morality considered by Korean mothers.	
	Degeling et al (2020) [19]	Australia		BMJ Open		1. The Australian general population 2. n=2008 3. Median 46 y (18-89 y) 4. 1015:993 5. Purposive sampling		Compare the value of core surveillance system attributes to the Australian public before and during the early stages of the COVID-19 pandemic		New technology raised concerns of privacy disclosure and misuse in the COVID-19 outbreak.		Quantitative; online survey		After the COVID-19 pandemic, participants demonstrated greater preference for a high data security surveillance system for public health.	
	Thomas et al (2020) [20]	Australia		JMIR Public Health Surveillance		Australians (excluding health care professionals or people who had been tested for COVID-19) n=1500 ≥18 y 1:1 Purposive sampling		Investigate the ethical issues of adopting the Australian government’s COVID safe app		App-based contact tracing for curbing the transmission of COVID-19 needs widespread adoption.		Quantitative; online national survey		Privacy, data storage, and technical functions are ethical issues that hinder contact-tracking apps.	
	Degeling et al (2019) [21]	Australia		Health Research Policy and Systems		1. Australian-based policy makers and experts in infectious diseases, epidemiology, food safety, health informatics systems, and health and technology law 2. n=44 3. N/A 4. N/A 5. Purposive sampling		Identify ethical issues in the adoption and effective implementation of a digital surveillance tool		Early detection of infectious disease outbreaks involves lack of social license or ethical and legal considerations.		Mixed method; online survey; framework analysis		Infectious disease monitoring systems raise issues such as personal privacy, forensic risks, potential unintended consequences, and the weakening of public trust.	
**Reports (n=24)**															
	Sweeney (2020) [22]	United Kingdom		Nature Machine Intelligence		N/A		Balance protecting public health with safeguarding civil rights regarding contact-tracing apps		Contact-tracing apps were used in COVID-19 surveillance but were less understood by people.		Discussion paper		Contact-tracing apps debate on protecting public health with safeguarding civil rights.	
	Gilbert et al (2019) [6]	Australia		Asian Bioethics Review		N/A		Highlight the urgency of having an ethical framework to guide the use of new technologies in communicable disease surveillance and control		There is considerable public opposition to allowing public health authorities access to personal health data for infectious disease surveillance.		Discussion paper		Informed public discussion, greater transparency, and an ethical framework will be essential to build public trust in the use of new technology for communicable disease control.	
	Schwalbe et al (2020) [23]	United States		Lancet		N/A		Artificial intelligence (AI) use in low- and middle-income countries		AI-driven intervention research in global health has less addressed ethical, regulatory, or practical considerations.		Discussion paper		Addressing privacy and security in digital development involves careful consideration of which data are collected and how data are acquired, used, stored, and shared.	
	Garattini et al (2019) [2]	United Kingdom		Philosophy & Technology		N/A		Provide a moral foundation for the societal acceptance and responsible development of technological advancement		There are many ethical impacts when applying BDA in infectious diseases.		Discussion paper		Automation and algorithmic reliance impact freedom of choice; BDA complexity impacts informed consent; reliance on profiling impacts individual and group identities and justice/fair access; and increased surveillance and population intervention capabilities impact behavioral norms and practices.	
	Parker et al (2020) [3]	United Kingdom		Journal of Medical Ethics		N/A		Outline ethical considerations in the deployment of digital surveillance systems for public health response		Mobile phone contact-tracing apps have raised many ethical questions in the COVID-19 pandemic.		Discussion paper		Privacy, liberty, responsibilities, data management, public trust and confidence, equity, fairness, justice, and data consistency need to be addressed in the deployment of mobile phone apps.	
	Katapally (2020) [24]	Canada		Journal of Medical Internet Research		N/A		Outline an evidence-based global digital citizen science policy, which provides a theoretical and methodological basis for ethically sourcing big data from citizens to tackle pandemics such as COVID-19		A cohesive societal effort with citizens’ full support is needed in pandemics.		Discussion paper		One of the biggest ethical challenges is data privacy and security. Individuals’ rights to privacy and anonymity through advanced encryption and secure server storage processes, informed consent, the ability to dropout and delete their own data, and data co-ownership, should be priorities.	
	Mbunge (2020) [25]	Eswatini		Diabetes & Metabolic Syndrome: Clinical Research & Reviews		N/A		Analyze the potential opportunities and challenges of integrating emerging technologies, including 5G technology, AI, and big data, into COVID-19 contact tracking		Contact-tracing technologies have limitations when used in the COVID-19 pandemic.		Literature review		Ethical or legal challenges might be socioeconomic inequalities in developing counties; security risks such as data security, confidentiality, integrity, and data availability of patients and contacts in COVID-19; the privacy issues of patients, which may lead to mental health problems; consent and voluntariness; and discrimination.	
	Garg et al (2020) [26]	India		JMIR Public Health and Surveillance		N/A		Describe Aarogya Setu, a first-of-its-kind participatory disease surveillance initiative in India and its ethical considerations		Opt-in, data integrity, and ethical concerns need to be addressed when using the new system for the COVID-19 pandemic.		Case report		The main ethical dilemma is how to ensure data protection and proper ethics while obtaining the benefits of public health surveillance, and how to ensure the ethical use of collected data and protect individual privacy.	
	Denecke (2017) [27]	Switzerland		Life Sciences, Society and Policy		N/A		Highlight the ethical issues that should be considered when integrating digital epidemiology with current practice and develop an ethical assessment model for digital disease detection (DDD) technologies		Usage of digital surveillance in epidemiology has different kinds of challenges.		Discussion paper		The model developed in this study might help to make aware the ethical aspects already in the development process, and possibly address them.	
	Kostkova (2018) [28]	United Kingdom		Life Sciences, Society and Policy		N/A		Outline 3 major ethical and governance challenges for digital epidemiology in the 21st century		Digital surveillance has created ethical, political, and legal challenges in infectious disease control.		Commentary report		Some of the ethical challenges of sharing data across various early warning tools to support risk assessment are ownership of personal data, transparency and clarity of public health data sharing, strong transparent disclosure, data privacy and security, and the balance between data sharing, personal data protection, stakeholder needs, and public good.	
	Vayena et al (2015) [29]	Switzerland		PLoS Computational Biology		N/A		Identify key ethical challenges associated with DDD activities and outline a framework for addressing them		DDD has many ethical challenges in infectious disease pandemics.		Discussion paper		The ethical challenges of DDD can be divided under 3 heads: context sensitivity (privacy and contextual integrity, transparency, and global justice); nexus of ethics and methodology (risk of harm, use of resources, trust, transparency, accountability); and legitimacy requirements (shared code of practice, mechanism for quick response to inaccuracies, addressing harms caused by DDD activities, common good).	
	De Jong et al (2019) [30]	Belgium		Emerging Infectious Diseases		N/A		Mitigate the ethical concerns of movement mapping of potentially infected persons		Mapping the movements of potentially infected persons has ethical challenges.		Discussion paper		Ethical obstacles are privacy in relation to the principles of autonomy and nonmaleficence; and a balance between costs, risks, and benefits for participants and communities in relation to the principles of beneficence and justice, such as stigmatized community, data withholding, and whether and how to communicate information on hotspots to the general population.	
	Kind (2020) [31]	United Kingdom		Patterns		N/A		Examine societal, political, legal and ethical perspectives on symptom tracking, contact tracing, and immunity		The UK government asked for more information to decide the use of technology in the COVID-19 pandemic.		Rapid evidence review		The ethical issues of digital contact tracing are human rights and data protection, inequalities, data quality limitations, false reporting risks, and centralization of large amounts of personal data.	
	Park et al (2020) [32]	Korea		JAMA		N/A		Identify ethical concerns over privacy involving the information technology–based tracing strategy in response to COVID-19		South Korea extensively used digital tools for tracing COVID-19 patients.		Discussion paper		Privacy controversies might unveil or infer embarrassing personal details, unwanted privacy invasion, public disdain, uneven scope, and granularity of disclosures by municipal and local governments.	
	Fraser et al (2020) [33]	United Kingdom		University of Oxford		N/A		Minimize the invasion of privacy by using digital contact tracing		Contact tracing was used to assist people in receiving warnings about COVID-19.		Discussion paper		The ethical issues include sensitively and specifically identifying infectious individuals, user uptake and adherence, notification, integration with local health policy, and ability to evaluate effectiveness transparently.	
	Cho et al (2020) [34]	Singapore		ArXiv Preprint		N/A		Discuss ways of ameliorating privacy concerns without decreasing the usefulness of contact-tracing apps		The Singaporean government released a mobile phone app to assist in tracking down exposures to COVID-19 patients, but there were privacy implications.		Discussion paper		Privacy is a central feature of conversations around mobile contact-tracking apps. Some privacy trade-offs can be endured for public health.	
	Klenk et al (2020) [35]	Netherlands		Ethics and Information Technology		N/A		Identify factors that pose a risk for fair group composition		Digital tracing technologies for COVID-19 control were reported to have ethical risks.		Discussion paper		Digital tracking apps will introduce new psychological, social, economic, and political risks.	
	De Montjoye et al (2020) [36]	United Kingdom		Computational Privacy Group Blog		N/A		Propose 8 questions to assess privacy in contact-tracing apps.		A contact-tracing app was developed to assist with COVID-19 control, and record location or close contact data.		Discussion paper		Privacy protection should rely on mathematical proof, and mitigation strategies should be considered only when necessary. We should focus on privacy and ensure security.	
	Bernier et al (2015) [37]	Canada		University of New Brunswick Law Journal		N/A		Highlight the personal privacy in electronic public health surveillance systems		Data surveillance has become a key component of pandemic response plans.		Discussion paper		The privacy governance framework is incomplete in ensuring the effective and protective use of personal information in response to epidemics.	
	Ienca et al (2020) [38]	Switzerland		Nature Medicine		N/A		Identify ethical issues when using digital surveillance systems in COVID-19		The COVID-19 emergency has used much more digital tools than previous outbreaks globally.		Discussion paper		Best practices should be identified to protect privacy and public trust.	
	Yasaka et al (2020) [39]	United States		JMIR mHealth and uHealth		N/A		Develop an effective contact-tracing smartphone app that respects user privacy by not collecting location information or other personal data		Smartphone-based contact tracing has been used in the COVID-19 pandemic to limit disease transmission.		Discussion paper		Users may be uncomfortable with applications that track real-time locations.	
	Barbieri et al (2020) [40]	Italy		Istituto Affari Internazionali		N/A		Discuss the ethics of technological solutions to mitigate COVID-19		Technological solutions to mitigate the COVID-19 crisis have been implemented in China and South Korea.		Discussion paper		In a pandemic crisis, the balance between privacy and public health tends to tilt toward the latter. However, a strong legal framework should be established around any such data-driven policy, taking into account the transition to “postepidemic” life.	
	Chan et al (2020) [41]	United States		arXiv		N/A		Improve the privacy and anonymity standards of mobile contact tracing		The COVID-19 pandemic has been controlled by large-scale adoption of contact tracing.		Discussion paper		Ethical issues of privacy protection, transparency, and reidentification risks of anonymous information.	
	Peter (2020) [42]	Australia		The Guardian		N/A		Discuss the acceptability of a coronavirus tracing app by Australians and how to implement tracing technology successfully		The Australian public is seeking a way to manage the COVID-19 pandemic.		Discussion paper		The ultimate success of tracking technology will depend on confidence and mutual respect. The ultimate test of any tracking technology will be the strength of the relationship between the public and the government.	

^a^N/A: not applicable.

### Ethical Issues

Textbox 1 shows the key ethical issues identified in the review summarized and clustered under individual, organizational, and societal levels with key themes presented under the corresponding concepts. Six domains, namely awareness of implementing digital infectious disease surveillance, digital integrity, trust, privacy and confidentiality, civil rights, and governance were highlighted.

**Ethical issues in utilizing artificial intelligence–augmented infectious disease surveillance systems based on the ethical framework of Asadi et al [15].**

**Individual level**

Data ownership

- Digital infectious disease surveillance systems challenge data ownerships rights
- Impacts of data ownership rights on public participation in digital infectious disease surveillance

Data control

- Inappropriate reidentification, sharing, or processing of personal information of infectious disease patients

Awareness

- Lack of understanding of data collection, access, processing, sharing, and storage of infectious disease surveillance systems
- Lack of and inability to give consent when enrolled into infectious disease surveillance systems

Trust

- Data governance, security, and data set bias undermines public trust
- Public mistrust in the necessity and effectiveness of contact-tracing technology for infectious diseases
- Public mistrust in governments’ strategies in using digital infectious disease surveillance

Privacy

- Concerns of privacy risks and allowance to infectious disease control
- Privacy risks on contact tracing for infectious diseases and social networks
- Disclosure of infectious disease information leads to business depletion, privacy invasion, and public disdain
- Necessity of infectious disease data anonymization and risk of reidentification
- Privacy and appropriate authority oversight on artificial intelligence (AI)-augmented infectious disease surveillance systems

Self-determination

- Autonomy/personal liberty to participate in and use AI-augmented infectious disease surveillance systems

Fear

- Fears of privacy violation, institutional control/penalties, and discriminatory/stigmatized effects when using AI-augmented infectious disease surveillance systems

**Organizational level**

Data quality

- Data accuracy, validity, veracity, and integrity of AI-augmented infectious disease surveillance systems
- Consequences of low data quality in AI-augmented infectious disease surveillance systems

Data sourcing

- Vulnerable populations/key demographics underrepresented in data sourcing could lead to invalid infectious disease control

Data sharing/disclosure

- Data sharing/disclosure for unethical purpose when using AI-augmented infectious disease surveillance systems
- Data disclosure needs clear standards and safeguards when using AI-augmented infectious disease surveillance systems

Algorithmic decision making

- Reliability, validity, and consequences of algorithmic decision making for infectious disease outbreaks
- Vulnerability of machine learning processes for infectious disease surveillance

Presentation

- Needs for transparent and clear presentation of algorithms, data processing, and hotspots of infectious diseases

Ethical capability

- Lack of ethical training on data collection and disciplinary measures on poor data quality for infectious disease surveillance

Ethical culture

- Consideration of cultural contexts in data collection, processing, and decision making for infectious disease surveillance

Ethical governance

- Transparency of digital infectious disease surveillance systems
- Lack of common technical or ethical standards for data usage in infectious disease surveillance
- Lack of an ethical governance framework to regulate algorithms, data collection, use, and management for infectious disease surveillance

**Societal level**

Power

- Imbalanced power relations among decision makers, researchers, and citizens when using AI-augmented infectious disease surveillance systems
- Unequal allocation and distribution of resources and benefits when using AI-augmented infectious disease surveillance systems

Social awareness

- Social awareness in purpose, risks and benefits, and consequences when setting up infectious disease surveillance programs

Surveillance

- Ethical surveillance with full public engagement and incentives when using AI-augmented infectious disease surveillance systems
- Ensure infectious disease surveillance systems protect civil liberties and rights

Principles and guidelines

- Lack of legislation and guidelines for infectious disease outbreaks and protection of data security, individual privacy, and discrimination

Authority

- The possibility of data misuse by authorities/agencies without legal authorization

Climate

- Digital infectious disease surveillance in a social environment leads to social stigma, discrimination, rumors, and prejudice

#### Awareness of Implementing Digital Infectious Disease Surveillance

The informants reported insufficient understanding at every stage of data collection and distribution of digital infectious disease surveillance systems. They felt they were not informed of their rights to refuse or their ability to withdraw consent. Irresponsible decision making was identified by infectious disease patients in relation to insufficient information provided about contact tracing technology. At the societal level, digital infectious disease surveillance based on BDA was poorly accepted by the general public due to their uncertainty about its purpose and the risks posed from the potential mitigation of data sharing. Consequently, they wanted more information about the digital infectious disease surveillance systems and wanted their associated ethical concerns and consequences addressed. They also expected organizations to make public and transparent the algorithms and data processes used, and use plain language when explaining infectious disease surveillance. Simultaneously, both the general public and field experts emphasized that governments or institutions should convey the importance of infectious disease outbreak control to communities without violating ethical principles.

#### Data Integrity

Data integrity weaknesses were identified as common in BDA. Unreliable or invalidated data sourcing or algorithms were seen to lead to inaccurate identification of outbreaks or infected individuals, false predication of an event’s trajectory or the likelihood of reoccurrence, and inaccurate notifications. The outcomes, such as inadequate data integrity, were further seen to continuously intensify economic losses to trade, tourism, and health services, causing unnecessary panic and the loss of public trust in health authorities. Some organizations and experts also worried that digital infectious disease surveillance systems would widen existing health care disparities or digital divides by underrepresenting vulnerable and at-risk populations such as older adults, children, and people in economically underresourced areas; for instance, data of COVID-19 hotspots influenced the allocation and distribution of resources [25]. It was recommended that digital infectious disease surveillance applications should consider ethical requirements and the rights of people from diverse regions and communities.

#### Trust

Some people questioned the necessity and effectiveness of using contact-tracing technologies in an AI-assumed situation. They mistrusted the AI-augmented systems’ capacity to send correct notifications to infected individuals to instruct them to quarantine during an infectious disease outbreak in a timely manner. Emerging technology risks, such as data breaches or data set biases and government strategies of mandatory application of digital contact tracing, could further undermine public trust. Individuals and relevant experts called for an open debate or scrutiny, transparent procedures for data usage, and public consultation plans and privacy regulations.

#### Privacy and Confidentiality

Digital infectious disease surveillance was identified to pose considerable risks to an individual’s rights to privacy and confidentiality. Contact tracing that linked a potential infectious disease with patients’ movements, locations, or social networks was seen as a considerable threat to further disclosure of sensitive information. For example, personal social interactions and contact history, especially in the case of sexually transmitted infections or HIV, could be revealed. Individuals were concerned that third parties or malicious users might access large health data sets for profit and/or abuse. Disclosure of private information was seen to lead to business depletion, privacy invasion, and public discrimination and stigmatization. Data anonymity, robust encryption processes, and deidentified aggregate data were contended to be crucial to all data privacy and security procedures in compliance with data protection regulations.

#### Civil Rights

Data ownership rights for public health surveillance were regarded as a major ethical challenge. Some reported that authorities or institutions limited their rights to decide the adoption of digital infectious disease contact tracing or surveillance systems. Personal liberties were also impacted by movement mapping, cross-border sharing of personal health information, and frequent security checks using QR codes for filling in personal information. Countries using centralized contact-tracing apps and privacy-by-design apps were seen to have the potential to impose restrictions on civil liberties, which, in turn, impacted ethical engagement in digital health. Data co-ownership and strengthening of transparency were seen as helpful to encourage individuals to participate in data visualization, analysis, and knowledge translation, and balance the power dynamics among decision makers, researchers, and citizens.

#### Governance

Technical and ethical standards, as well as legislation and guidelines for infectious disease outbreaks, data security, protection of individual privacy, and avoidance of discrimination were considered poorly developed and incomplete. Technology companies were recommended to establish a mechanism to deal with inaccurate epidemic reporting and dissemination of misinformation, and were expected to develop rapid ethical assessment, training, and disciplinary measures for data collection or sharing. Governance institutions or bodies, such as national health commissions, medical councils, and company boards, were further recommended to provide appropriate oversight on the performance of algorithms and data usage. Some literature argued that an independent privacy audit was needed to secure a transparent approach for the public [33,36]. Both individuals and relevant experts also supported data sharing protocols to specify the scope and granularity of disclosure. An example provided was that personal names should not be publicly available, and personal information should only be gathered and shared within a period of time regulated by law and clearly justified based on population health needs.

## Discussion

### Challenges of Using AI Surveillance Systems for Infectious Diseases

The emergence of AI surveillance systems for infectious diseases promises tangible global public health benefits, but these are accompanied by significant ethical, political, and legal challenges, which span over a wide spectrum on 3 levels. Six main themes were generated from this systematic review, ranging from people’s awareness and knowledge of digital infectious disease surveillance systems and personal privacy on the individual level to organizational issues of maintaining data integrity and security, and the lens was extended upward to the societal level, involving public trust, civil rights, and the need for a governance framework with ethical oversights. Some of the challenges are inherent to public health practice and only heightened by the use of digital tools, and others, such as public trust and awareness of digital surveillance, algorithmic decision making, and data security, are specific to AI approaches and largely unprecedented. It is vital to consider these challenges to enhance individuals’ rights, privacy, public responsibilities, and optimal population health outcomes so that digital surveillance can tackle pandemics ethically.

### Strengths and Limitations

Studies and position papers on the ethical implications of AI surveillance for infectious diseases have gained momentum since 2019 in response to the outbreak of the COVID-19 pandemic; the dearth of studies prior to this makes it challenging to reveal any time trends. Furthermore, most articles included in the review were discussion papers, limiting the ability to evaluate generalizability, transferability, and rigor or multiple stakeholders’ perspectives on the use of AI-augmented infectious disease surveillance. The 5 empirical studies had restricted sampling approaches both in terms of size and stakeholder representation (health professionals and community groups), with 4 of the studies being conducted in Australia alone. Few papers involved vulnerable or diverse populations, and none involved specific cultural or socioeconomic groups, further limiting the scope of the review. Furthermore, articles acknowledged the inherent strengths and weaknesses of the public health system operating at the location of the study.

### Retributions Versus Common Good for Public Health and Long-term Impacts

The tensions between human and civil right discourses and the need for rapid public health responses are exacerbated with the use of BDA/AI in the context of a pandemic [2]. There are medicolegal and moral retributions and concerns arising from utilizing available epidemiological information through a highly effective and responsive infectious disease surveillance method that could protect local and, potentially, global communities from serious infectious disease outbreaks [3,18]. Conversely, routine collection and linkage of detailed personal information pose considerable risks for the violation of individual and civil freedoms of choice and privacy [19].

As of July 21, 2021, there have been 191 million confirmed cases and 4.11 million deaths globally from COVID-19 [43]. Given that sound public health interventions are predicated upon promoting and protecting the health of communities, timely, cost-effective, and socioculturally informed primary care interventions, advocacy, and empowerment with long-term impact evaluations are required. There are strong reasons to develop an ethical governance framework to support AI-augmented infectious disease surveillance to achieve these outcomes. The question of how to balance what is needed for the “good of public health outcomes” and human rights in pandemic crisis situations is highlighted in the literature reviewed [33,37].

### Trust Building and Privacy Protection

Detection and notification of infectious disease outbreaks requires prompt accurate disease diagnosis and follow-up of infected individuals and their close contacts. An early AI-augmented warning system has exponential potential for implementing real-time, responsive, and adaptive calculations. This means that substantial personal information, such as names, ages, locations, and relevant heath data, will be accessed in a timely manner and collected by AI systems for calculation, analysis, and notification. Public opt-in and well-founded trust in the digital system, its implementation, and the governance framework are therefore essential factors regarding ethical issues at the organizational and societal levels for infectious disease surveillance, which could enable the acceptability and effectiveness of the system.

Even with optimal technical standards to maximally reduce the risks and consequences of data misuse, data safety, security, and integrity cannot be guaranteed. The role of AI-augmented infectious disease surveillance might only be supplementary for public health, while ethical issues are carefully observed. One of the most important challenges facing AI is to design and develop appropriate methods to deidentify personal information and protect privacy, yet a greater risk of false positive and false negative notifications exists with higher-level deidentified data [33].

Moreover, well-founded trust and confidence vary in different countries and between individuals. People in democratic countries tended to distrust AI-augmented surveillance systems by challenging them when personal information is reported to health authorities, often without appropriate informed consent [3]. In contrast, Chinese citizens expressed their trust in the Chinese government’s response to COVID-19, which they felt had been highly successful in controlling the spread of the virus through the use of mobile phone data combined with intensive testing and restriction programs [44]. Although there is no unified standard to establish trust, the need for effective, transparent, accountable, and independent oversight is very important.

### Generation of an Ethical Framework and Global Governance Structures

Digital standards and guidelines for developing and evaluating the performance of infectious disease surveillance alone are insufficient. The scope of the ethical framework in Textbox 1 needs to be expanded globally. Cross-national and national governance structures; institutional systems with regulatory, medical, ethical, and legal frameworks; and benchmarking standards have essential roles to play in the development and deployment of these new health technology systems. However, this review identified the urgent need for an ethical framework to underscore all AI-augmented infectious disease surveillance systems [18,19,30]. Considering the rapid development of global trade supply chains, mass gatherings, and international travel, World Health Organization’s International Health Regulations in 2005 [45] outlined the cross-border implications of a pandemic response and provided a framework for sharing, monitoring, and evaluating information from the sources of infections [37]. In response to a pandemic emergency, cross-border sharing of personal health data is essential for tracing infectious disease patients and their contacts. At the same time, cross-border sharing further raises the heightened and unique risk for individual privacy and security breaches. In turn, a spectrum of actions regarding ethical, political, and legal implications must be framed within strict safeguards and needs to be mandated globally [37].

### Implications

A systematic medical, ethical, and legal framework is necessary for governance of AI-augmented infectious disease surveillance and the protection of personal privacy and data integrity. Public health systems should maximally increase the social awareness of AI surveillance and BDA for infectious diseases, and implement new technologies for infectious disease surveillance in a more person-centered and humane manner. Future research needs to focus on the setting up and implementation of an AI-augmented infectious disease surveillance system underscored by an ethical framework based on universal human rights. Decision makers should take into account varying and diverse population needs, sociocultural status, and regulatory and legal governance in order to promote trust building between end users, including infectious disease patients, doctors, and AI system implementers. This systematic review is intended to contribute to the development of a more comprehensive and concrete ethical framework for AI-augmented infectious disease surveillance, which will enable it to ultimately maximize public health responsiveness synergized within an ethical context.

## Appendix

Multimedia Appendix 1 PRISMA (Preferred Reporting Items for Systematic Reviews and Meta-Analyses) checklist.

## Abbreviations

AI: artificial intelligence
BDA: big data analytics
EHR: electronic health record
ML: machine learning
